# Pharmacokinetics and Safety of Icotinib Hydrochloride Cream in Patients with Mild to Moderate Chronic Plaque Psoriasis: A Randomized Double-Blind Vehicle-Controlled Phase 1 Study

**DOI:** 10.1155/2019/9072683

**Published:** 2019-05-02

**Authors:** Lunfei Liu, Honggang Lou, Jiong Zhou, Ying Shen, Min Zheng, Zourong Ruan

**Affiliations:** ^1^Department of Dermatology, The Second Affiliated Hospital, Zhejiang University School of Medicine, China; ^2^Department of Dermatology, The Fourth Affiliated Hospital, Zhejiang University School of Medicine, China; ^3^Center of Clinical Pharmacology, The Second Affiliated Hospital, Zhejiang University School of Medicine, China

## Abstract

**Objective:**

This phase I study aimed to systematically assess the safety, local tolerability, pharmacokinetics, and preliminary efficacy of topical icotinib hydrochloride cream in patients with mild to moderate plaque psoriasis.

**Materials and Methods:**

Eligible Chinese adult patients with mild to moderate psoriasis were assigned to the icotinib cream or vehicle group. Icotinib cream with increasing concentrations (0.5%, 1.0%, 2.0%, and 4.0%) or vehicle were administered by the fingertip unit method to the skin lesions twice a day for 4 weeks. Safety assessments included the incidence and severity of adverse events (AEs), local tolerability at the treatment area, vital signs, and laboratory examinations. Plasma levels of icotinib were also measured for the pharmacokinetics calculation. The efficacy was preliminarily explored by assessing the improvement in the severity level using Target Plaque Severity Score (TPSS) and overall improvement using the Psoriasis Area Severity Index (PASI) and Dermatological Quality Life Index.

**Results:**

Forty-one patients were enrolled and qualified for safety analysis. 27 (65.9%) patients experienced at least one AE, of which application-site adverse drug reactions (ADRs) were reported in 6 (14.6%) patients. All ADRs were of grade 1 or 2, most common irritation (4.5%), itching (3.1%), and erythema (2.4%), and resolved during follow-up. The systemic exposure to icotinib was very low; the highest plasma concentration was 0.214 ng/mL, while the area under the curve from 0 to 12 hours was 1.626 h·ng/mL. The TPSS improved for all icotinib groups after treatment in a dose- and time-dependent manner.

**Conclusion:**

This phase 1 study demonstrated favorable safety, tolerable toxicity, and preliminary efficacy of icotinib cream in patients with mild to moderate psoriasis. The dose concentration of 2.0% (twice daily based on the fingertip unit method) is recommended for further study.

**Study Design:**

This is a single-center, randomized, double-blind, and vehicle-controlled study.

## 1. Introduction

Among the most baffling and persistent skin disorders, psoriasis mainly occurs in young adults, can affect the entire body, and does not differ in prevalence between men and women [1, 2]. Although the epidemiological data of psoriasis in Asians showed a lower incidence than those in Europeans and North Americans [3, 4], the treatment of psoriasis has become increasingly important in Asians in recent years. Epidemiologic studies reported a prevalence of psoriasis of 2–4% in Western populations [5] versus 0.47% in China [6]. The clinical manifestation of psoriasis is raised well-demarcated erythematous oval plaques with adherent silvery scales. Pathologically, psoriasis is mainly identified by hyperproliferative epidermis with premature keratinocytes and parakeratosis. In contrast to that of normal skin, the psoriatic microvasculature is characterized by tortuous and leaky blood vessels that facilitate leukocyte migration into inflamed skin [7]. The disease has a long course and tends to recur, with some cases remaining almost unhealed over a lifetime, creating a lifelong burden for patients.

The pathogenesis and molecular biological mechanism of psoriasis have not yet been fully clarified [8–10]. Nevertheless, multiple factors such as genetic predisposition, life style, psychophysical traumas, radiation, and infection have been postulated to be triggers to this skin disease [11]. Currently, most psoriasis treatment drugs focus on blocking the formation and expression of different pathogenic factors and their receptors in the postulated pathogenesis of psoriasis. This “free combat” strategy has not completely hit the key self-control mechanism for pathophysiological disorders of psoriasis. The first-line management of mild to moderate psoriasis involves topical treatment [12]. Systemic therapy and phototherapy are used to treat moderate to severe psoriasis and often supplemented with topical therapies [13].

Epidermal growth factor receptor (EGFR) plays a critical role in the growth and proliferation of epidermal cells and participates in the excessive proliferation and differentiation of psoriatic keratinocytes [14, 15]. Besides, the downstream signal molecules of EGFR, including the Erk, Akt, and Stat families, are expressed and phosphorylated at significantly higher levels in psoriasis lesions than in nonlesioned areas or normal skin [16, 17], which indicates that the downstream signals of EGFR were also closely related to the development of psoriasis. Therefore, inhibitors with potent activity blocking signaling pathways of EGFR may have the potential to treat psoriasis.

Icotinib is a small-molecule EGFR tyrosine kinase inhibitor [18–20]. Preclinical studies [14] showed that icotinib hydrochloride could specifically inhibit EGFR tyrosine kinase and block the activation of downstream signaling pathways of Stat3 and Akt, regulating the proliferation and differentiation of keratinocytes and inhibiting angiogenesis and further improving the pathological conditions of epidermal keratosis and fine epidermis in psoriasis. A previous phase I clinical study (NCT02574091) demonstrated that icotinib cream at 1% and 2% concentrations was well tolerated by both healthy subjects and psoriasis subjects. In addition, symptom improvements were observed in subjects with mild to moderate psoriasis.

This single-center randomized double-blind vehicle-controlled study assessed the pharmacokinetics, safety, and preliminary efficacy of icotinib hydrochloride cream in patients with mild to moderate chronic plaque psoriasis.

## 2. Patients and Methods

The study was performed in accordance with the Declaration of Helsinki and the principles of Good Clinical Practice. The protocol was approved by the ethics committee of the 2^nd^ Affiliated Hospital School of Medicine, Zhejiang University, and registered at www.clinicaltrials.gov (NCT02801435). Written informed consent was obtained from all participants.

### 2.1. Patients

#### 2.1.1. Main Inclusion and Exclusion Criteria

Eligible patients were aged 18–65 years with a clinical diagnosis of plaque psoriasis for at least 6 months; plaque psoriasis should cover less than 10% of the total body surface area (BSA); the affected area on the limb and/or trunk should be ≥1% of the BSA. Each participant had a target plaque area (TPA) ≥ 9 cm^2^, Target Plaque Severity Score (TPSS) ≥ 5, and induration subscore ≥ 2. Male participants should be ≥50 kg and female participants should be ≥45 kg; body mass index should be 19–28 kg/m^2^. Patients were excluded if they had nonplaque forms of psoriasis, received underlying treatments before the first study dose, or were evaluated by investigators as unsuitable for this study.

#### 2.1.2. Dosage

Patients were randomized at ratio of 8:2 to receive icotinib cream or vehicle (blank cream with identical appearance/taste to those of icotinib cream; Betta Pharmaceutical Co., Ltd., Hangzhou, Zhejiang, China). Therapeutic drug doses were 0.5%, 1.0%, 2.0%, and 4.0% in the dose-escalation study. Icotinib cream and vehicle were prepared by the investigator according to the 2% of BSA per fingertip unit and administered twice daily. On each visiting day, the treatment area was not moisturized within 24 hours before the assessment.

#### 2.1.3. Safety Assessment

Safety assessments included the incidence and severity of adverse events (AEs) including an itching/burning sensation, skin irritation reaction (4-point scale: none, mild, moderate, and severe) at the treatment area, vital signs, and laboratory examinations.

#### 2.1.4. Pharmacokinetics

Plasma concentration-time data and pharmacokinetic parameters were examined. Sampling points were as follows: 30 min before treatment and 2 h, 4 h, 8 h, and 12 h after administration on the first and last day, and before dosing at day 8, day 15, and day 22. At each point, 2.5 mL of venous blood was collected from a forearm vein and placed immediately in a tube containing EDTA-2K anticoagulant and centrifuged at 3000 rpm for 10 min at 4°C. The plasma was stored at -70°C. The plasma concentration-time data of each subject were calculated using a pharmacokinetic test and the concentration-time curve was plotted. The pharmacokinetic parameters with mean and standard deviation were calculated simultaneously. Pharmacokinetic parameters included maximum observed concentration (C_max_), time to C_max_ (T_max_), and area under the concentration-time curve (AUC_0-t_).

#### 2.1.5. Efficacy

During the TPSS evaluation, a single target plaque not less than 9 cm^2^ in arealocated on the trunk or limbs was selected at baseline. Dermatological clinical evaluations were conducted by experienced dermatologists and the same investigator for each patient. The target plaque was assessed separately for erythema (E), scale (S), and thickness (T) using a 5-point severity scale (0, none; 1, slight; 2, moderate; 3, marked; 4, very marked), and the scores summed to produce the TPSS sum score (13-point sacle; maximum [most severe] score, 12) [21]. The overall improvement was evaluated through the PASI using the calculation formula (prePASI –afterPASI)/prePASI × 100%. PASI50, PASI75, and PASI90 mean improvement degrees of 50%, 75%, and 90% are achieved, respectively. The Dermatological Quality Life Index (DLQI) was adopted to assess patients' quality of life, including symptoms and feelings, daily activities, amateur activities, work or learning, interpersonal relationships, and therapy [22].

#### 2.1.6. Statistical Analysis

Tolerance studies were dominated by descriptive statistics. Qualitative indicators are described in terms of frequency, percentages, or composition ratios; quantitative indicators are described as mean ± standard deviation (SD) or maximum, minimum, and median. Variance analysis was performed on the differences between the clinical indicators and laboratory indicators before and after administration [23]. Wilcoxon's rank sum test was used to compare the groups in terms of the severity and improvement of target lesions. The compliance comparison of PASI50, PASI75, and PASI90 between groups was performed by Fisher's exact probability test. A paired t-test was used for the intragroup comparison of each visiting point relative to baseline for improved PGA value. The intragroup comparison of DLQI improvement score at baseline and each visiting point were performed using Wilcoxon's signed rank test.

## 3. Results

### 3.1. Patients

Between September 17, 2016, and June 8, 2017, a total of 41 patients were enrolled in the study. All patients were included in the safety and efficacy analysis sets except for one patient in the vehicle group due to a major protocol violation. The demographic data are listed in Table 1.

#### 3.1.1. Safety Analysis

A complete physical examination of all patients revealed no abnormalities before and after treatment. The laboratory examination indicated that alanine aminotransferase, aspartate aminotransferase, urinary leukocytes, and urinary red blood cells were occasionally abnormal, and all recovered to normal level without management. A total of 44 cases of adverse events occurred in 27 subjects during the study. In addition, there were 7 cases of ADRs in 6 subjects; the most common ADRs were irritation (4.5%), itching (3.1%), and erythema (2.4%). A correlation analysis revealed no correlation between plasma concentrations of icotinib and ADRs.

#### 3.1.2. Pharmacokinetics

**Figure 1:**
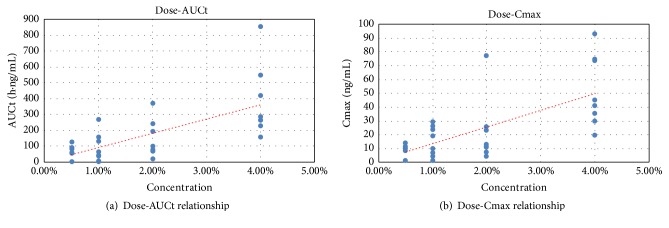
Correlation between concentration of drug preparation and system exposure after topical single administration.

Following a single dose, the plasma concentration of icotinib was not detected in some samples (the lower limit of quantification was 5 pg/mL). The highest plasma concentration was 0.214 ng/mL and the AUC_0-12h_ was 1.626 h·ng/mL. After 28 days of continuous drug administration, the systemic concentration increased, with the highest C_max_ value of only 3.95 ng/mL and the highest exposure AUC_*τ*_ of only 20.50 h·ng/mL. The concentration-time profile showed no absorption or elimination characteristics. The main pharmacokinetic parameters of single and multiple doses in each concentration group are summarized in Figure 1 and Table 2.

#### 3.1.3. Efficacy

**Figure 2:**
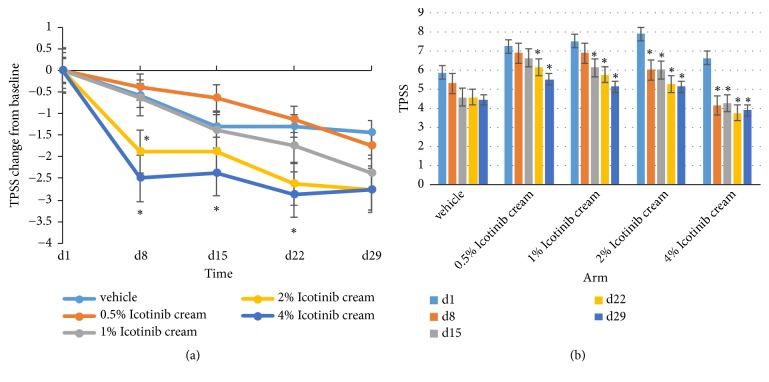
Target Plaque Severity Score (TPSS) change from baseline. (a) TPSS change in different arm, ^*∗*^*p* < 0.05, icotinib cream vs. vehicle; (b) TPSS in different arm, ^*∗*^*p* < 0.05 vs. baseline (d1).

As the treatment continues, there was a tendency toward an remission in severity of the target lesion as icotinib concentration increased, indicated by the TPSS in each dose group decreasing in a dose-dependent manner (Figure 2(a)). The improvement in the TPSS was more obvious in icotinib cream group than that in the vehicle group. Meanwhile, the improvement in the TPSS was much more considerable in the high-dose groups at day 8, 15, and 22. Moreover, patients in the 2% and 4% dose groups experienced prompt and durable remission initiated at day 8 (Figure 2(b)).

**Figure 3:**
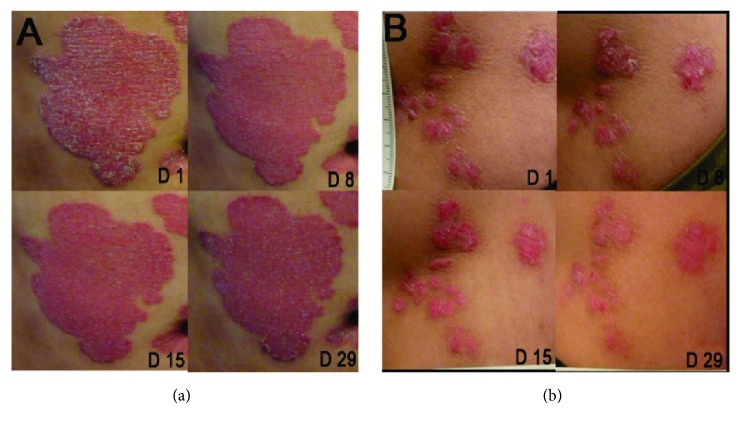
Target plaque photographs at baseline and weeks 1, 2, and 4 in a patient receiving vehicle (a) and 2% icotinib cream (b). Icotinib cream was applied to the left waist: Target Plaque Severity Score (TPSS) at baseline = 8, at week 4 = 3 (-62.5%). Vehicle was applied to the abdomen: TPSS at baseline = 7, at week 4 = 6 (-14.3%).

With increased dose administration, the total TPSS score of target lesion severity decreased and showed a dose-effect relationship. Patients in the high dose group (2% and 4%) derived more clinical benefits than those in other groups.  Figure 3 lists the target plaque change in a patient treated with vehicle (Figure 3(a)); the area of the targeted skin lesion treated with 2% icotinib cream (Figure 3(b)) reduced obviously. Maximum improvement was noted after 4 weeks of treatment.

#### 3.1.4. Degree of Infiltration Hypertrophy (Thickness, T) in Targeted Skin

**Figure 4:**
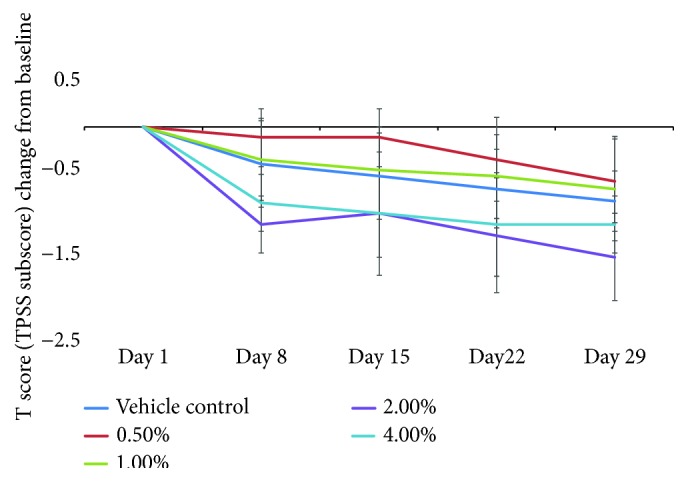
The T score (TPSS subscore) changes from baseline (d1).

The T score (TPSS subscore) changes from baseline were time dependent during weeks 1, 2, 3, and 4 (Figure 4). With the increase in medication time, the changes in TPSS subscore in each dose group gradually decreased and showed a dose-effect relationship; there was an increasing tendency of improvement with the increasing dose concentration. The improvement degree of target lesion severity score and its intragroup comparisons are shown in Tables 3 and 4.

#### 3.1.5. Overall Improvement

At day 28, PASI50 was achieved in 4 subjects (10.3%) after administration (1 subject in the 0.5% group, 1 subject in the 1% group, and 2 subjects in the 4% group). One case in the 4.0% dose group even achieved PASI75 on day 29, although no subjects achieved PASI90. As treatment time and dose concentration increased, PASI scores gradually decreased, with the 2% and 4% dose groups showing the most significant decline. The PASI scores of each dose group after drug administration are shown in Table 5.

#### 3.1.6. DLQI Changes

Most patients had a decreased DLQI score versus baseline at day 28 after administration: 5 in the 0.5% dose group, 2 in the 1.0% dose group, 3 in the 2.0% dose group, 2 in the 4.0% dose group, and 1 in the vehicle group. Among them, the decrease of DLQI score was up to 9 points (Table 6).

## 4. Discussion

Icotinib, a small-molecule EGFR tyrosine kinase inhibitor, is the first-line treatment for advanced NSCLC. Based on the fact that the EGFR signaling pathway plays an important role in the development of psoriasis [14, 15], here we reported the pharmacokinetics, safety, and preliminary efficacy of icotinib cream in Chinese psoriasis patients. Although the circulating concentration of icotinib increased in a dose-dependent manner, the pharmacokinetic data showed that the overall system exposure was still very low, which was even lower than 1% of a single oral dose of 100mg icotinib tablet (healthy subjects orally received 100 mg icotinib hydrochloride tablets for a single oral dose; related C_max_, 719 ± 206 ng/mL; AUC, 3361 ± 703 h.ng/mL). This finding indicates that only a small amount of icotinib cream enters the systemic circulation. Therefore, its systemic toxicity is extremely low.

Inconsistent with the safety profile of orally administered Icotinib tablets in NSCLC patients, the external use of icotinib cream was well tolerated in psoriasis patients. In this study, 65.9% of patients experienced AEs, with the erythema, itching, and irritation being the most common symptoms, and no systemic toxicity was observed. The incidence of AEs or ADRs was similar across treatment groups. All AEs and ADRs were mild or moderate and resolved during the observation or follow-up period. Additionally, there were no dose reductions or discontinuations due to AEs. This favorable safety was also supported by the results in our previous study in animal models, which showed that after the topical administration of icotinib cream the drug concentration in the skin is higher than that of the overall system [14]. The transdermal ability of icotinib is weak, which means that only a small amount enters the circulation. Taken together, although the favorable safety profile of icotinib has been confirmed in NSCLC patients who received icotinib tablets orally, we believe that the external use of icotinib in psoriasis patients is much safer.

Promising efficacy was also observed in the present study, and the TPSS improvement was observed in all icotinib groups after treatment in a dose- and time-dependent manner. Interestingly, compared with the concentration of 0.5% and 1%, the concentrations of 2% and 4% were well tolerated with comparable efficacy. Therefore, we believe that the icotinib cream at the concentration of 2% deserves further study. In addition to the EGFR signaling tracks postulated in the present study, clarifying whether other mechanisms such as cutaneous immunological homeostasis and cutaneous barrier's integrity are involved in the efficacy of topical icotinib cream will be interesting. Mattozzi et al. had reviewed the pathogenesis of psoriasis and suggested that preventing alterations of the immune homeostasis and restoring cutaneous barrier were associated with improvement in the clinical signs and symptoms of psoriasis or other chronic skin diseases. Moreover, vitamin D might play an important role in the treatment of psoriasis by maintaining the cutaneous barrier's integrity, keeping immune homeostasis, modulating the proliferation of keratinocyte, and regulating the microbial flora and the response of the host to skin infective diseases [11]. Furthermore, as combined therapies have been changing the landscape of treatment for many diseases, the favorable safety profile and preliminary efficacy of topical icotinib cream would be supportive for future exploration of potential combinations with other therapies, such as vitamin D supplementation. Psoriasis patients may get more benefits from combined topical icotinib cream treatment with other therapies.

In conclusion, icotinib cream had a favorable safety and pharmacokinetic profile in patients with mild to moderate chronic plaque psoriasis. The promising efficacy supports further development of this agent and the dose concentration of 2.0% (twice daily based on the fingertip unit method) is recommended for further study.

## Figures and Tables

**Table 1:** Demographic data of the patients in each group.

		Dose group					Total	*P* value
		0.5%	1.0%	2.0%	4.0%	Vehicle		
N		8	9	8	8	7	40	
Age (yr)	Mean ± SD	42.4 ± 7.93	36.6 ± 13.92	40.3 ± 12.8	41.8 ± 12.49	46.0 ± 14.74	41.2 ± 12.31	0.6689
	Median	41.5	31	37.5	43.5	52	41.5	
	Range	33-56	22-55	24-58	25-56	21-59	21-59	
Gender (M/F)		6/2	5/4	8/0	6/2	6/1	31/9	0.2999
Height (cm)	Mean ± SD	168.1 ± 6.16	164.5 ± 7.39	167.7 ± 5.55	165.0 ± 6.44	170.4 ± 4.84	167.0 ± 6.26	0.3406
	Median	169.5	164.4	168.0	162.5	172.0	167.8	
	Range	155-174	155-180	157-174	156-173	164-175	155-180	
Weight (kg)	Mean ± SD	67.74 ± 11.53	65.06 ± 10.55	64.06 ± 10.16	66.95 ± 10.51	67.67 ± 8.01	66.23 ± 9.86	0.9338
	Median	66.5	65.7	62.35	67	66.3	66.15	
	Range	55-84.1	49-79.3	53.5-79.5	46.4-80	56-78.6	46.4-84.1	
BMI (kg/m^2^)	Mean ± SD	23.9 ± 3.07	24.04 ± 3.63	22.76 ± 3.34	24.53 ± 3.17	23.39 ± 3.26	23.74 ± 3.19	0.8553
	Median	24.3	22.9	22.8	24.75	21.9	23.95	
	Range	19.3-27.8	19.1-28.7	19.0-28.2	19.1-29.7	19.8-27.8	19.0-29.7	

**Table 2:** Main pharmacokinetic parameters after single and multiple doses in each concentration group.

		C_max_ (ng/ml)			AUC_0-12h_ (ng*∗*h/ml)			T_max_ (h)		
Group	Administration	Average	Max	Min	Average	Max	Min	Median	Max	Min
0.5%	SD	0.039	0.069	0.006	0.300	0.635	0.012	9.97	12.03	4
	MD	0.340	1.220	0.026	3.168	9.080	0.284	2.01	11.98	0

1.0%	SD	0.050	0.107	0.005	0.349	0.780	0.032	6.03	12.02	2
	MD	0.337	0.680	0.149	2.570	4.927	1.057	2.04	12	0

2.0%	SD	0.051	0.154	0.016	0.340	0.741	0.097	6	12	2
	MD	1.165	2.910	0.146	7.426	20.494	0.922	4	12	0

4.0%	SD	0.099	0.214	0.023	0.735	1.627	0.172	8	12	4
	MD	1.737	3.950	0.426	9.152	13.928	2.828	2	8	0

**Table 3:** The degree of improvement in the total Target Plaque Severity Score at each assessment timepoint during treatment.

	Dose group					
	0.5% (N=8)	1.0% (N=9)	2.0% (N=8)	4.0% (N=8)	Vehicle (N=7)	*P* value
Day 8	4.91 (6.80)	6.94 (11.02)	23.49 (10.74)	38.11 (12.13)	8.36 (23.19)	0.0004
Day 15	8.56 (9.84)	15.28 (17.19)	20.06 (22.61)	36.33 (12.39)	20.41 (34.04)	0.0663
Day 22	15.33 (11.94)	21.88 (19.52)	29.96 (2.53)	43.48 (15.62)	21.23 (18.03)	0.0172
Day 28	23.89 (16.64) *∗*	31.25 (21.13)	31.81 (26.57)	41.99 (19.90)	24.56 (19.17)	0.4666

*∗*, *p* < 0.05 compared with baseline (day1).

**Table 4:** The assessment results based on Target Plaque Severity Score at each assessment timepoint during treatment.

		Dose group						
		0.50%	1.00%	2.00%	4.00%	Vehicle	Total	*P *value
		(N=8)	(N=9)	(N=8)	(N=8)	(N=7)	(N=40)	
	Cured	0	0	0	0	0	0	*p*=0.016
	Obviously effective	0	0	0	0	0	0	
Day 8	Effective	0	2 (22.2%)	4 (50.0%)	7 (87.5%)	3 (42.9%)	16 (40.0%)	
	No effect	8 (100.0%)	7 (77.8%)	4 (50.0%)	1 (12.5%)	2 (28.6%)	22 (55.0%)	
	Deteriorating	0	0	0	0	2 (28.6%)	2 (5.0%)	
	*Total*	8 (100.0%)	9 (100.0%)	8 (100.0%)	8 (100.0%)	7 (100.0%)	40 (100.0%)	
	Cured	0	0	0	0	0	0	*p*=0.138
	Obviously effective	0	0	0	0	1 (14.3%)	1 (2.5%)	
Day 15	Effective	1 (12.5%)	3 (33.3%)	4 (50.0%)	7 (87.5%)	3 (42.9%)	18 (45.0%)	
	No effect	7 (87.5%)	5 (55.6%)	3 (37.5%)	1 (12.5%)	1 (14.3%)	17 (42.5%)	
	Deteriorating	0	1 (11.1%)	1 (12.5%)	0	2 (28.6%)	4 (10.0%)	
	*Total*	8 (100.0%)	9 (100.0%)	8 (100.0%)	8 (100.0%)	7 (100.0%)	40 (100.0%)	
	Cured	0	0	0	0	0	0	*p*=0.196
	Obviously effective	0	0	0	1 (12.5%)	0	1 (2.6%)	
Day 22	Effective	3 (37.5%)	5 (62.5%)	7 (87.5%)	6 (75.0%)	4 (57.1%)	25 (64.1%)	
	No effect	5 (62.5%)	2 (25.0%)	0	1 (12.5%)	3 (42.9%)	11 (28.2%)	
	Deteriorating	0	1 (12.5%)	1 (12.5%)	0	0	2 (5.1%)	
	*Total*	8 (100.0%)	8 (100.0%)	8 (100.0%)	8 (100.0%)	7 (100.0%)	39 (100.0%) *∗*	
	Cured	0	0	0	0	0	0	*p*=0.475
	Obviously effective	0	0	1 (12.5%)	1 (12.5%)	0	2 (5.1%)	
Day 28	Effective	4 (50.0%)	5 (62.5%)	5 (62.5%)	6 (75.0%)	5 (71.4%)	25 (64.1%)	
	No effect	4 (50.0%)	3 (37.5%)	1 (12.5%)	1 (12.5%)	2 (28.6%)	11 (28.2%)	
	Deteriorating	0	0	1 (12.5%)	0	0	1 (2.6%)	
	*Total*	8 (100.0%)	8 (100.0%)	8 (100.0%)	8 (100.0%)	7 (100.0%)	39 (100.0%)	

**Table 5:** PASI scores at each assessment timepoint during treatment (mean (SD)).

				Dose group (%)		
	0.50%	1.00%	2.00%	4.00%	Vehicle	
	(N=8)	(N=9)	(N=8)	(N=8)	(N=7)	*P* value
Day 8	7.51 (1.942)	7.96(1.870)	6.51 (3.182)	4.45 (1.897)	4.90 (1.270)	
Day 15	7.43 (2.054)	7.32(1.914)	5.95 (2.941) *∗*	3.91 (1.816)	4.60 (2.022)	0.153
Day 22	6.78 (2.071) *∗*	6.69 (1.679)	5.60 (2.666) *∗*	3.45 (1.456) *∗*	4.71 (2.299)	0.04
Day 28	6.35(1.884) *∗*	6.11 (2.282) *∗*	5.96 (2.540)	3.54 (1.985)	4.33 (2.189)	0.009

*∗*, *p* < 0.05 compared with baseline (day 1).

**Table 6:** Dermatology Life Quality Index (DLQI) score (mean (SD)).

	Dose group (%)					
	0.5% (N=8)	1.0% (N=9)	2.0% (N=8)	4.0% (N=8)	Vehicle (N=7)	Total (N=40)
Day 8	-0.4 (2.67)	0.9 (1.54)	-1.4 (2.50)	2.1 (2.75)	2.3 (2.63)	0.7 (2.70)
Day 15	-0.4 (3.50)	1.3 (2.55)	-0.1 (2.47) *∗*	0.9 (3.18)	0.7 (4.03)	0.5 (3.06)
*P* value	1.0	0.5	*0.0313*	0.2813	0.5313	0.7258
Day 22	-0.3 (3.37)	0.5 (3.55)	-0.3 (1.67)	1.4 (3.25)	1.4 (3.87)	0.5 (3.13)
*P* value	0.9375	0.7188	0.125	0.375	0.625	0.9731
Day 28	-0.6 (4.37)	1.1 (3.27)	0.8 (2.38) *∗*	1.4 (2.92)	2.3 (3.68)	0.9 (3.34)
*P* value	1.0	0.8125	*0.0313*	0.4688	0.7188	0.3367

*∗*, *p* < 0.05 compared with the DLQI score at day 8.

## Data Availability

As for our research data, the summary of results data can be found in our manuscript; the detailed data used to support the findings of this study were supplied by the Second Affiliated Hospital, Zhejiang University School of Medicine, under license, and so cannot be made freely available. Requests for access to these data should be made to Data Storage Office of the Ethics Committee of the Second Affiliated Hospital.
